# Bone morphogenetic protein 2 (BMP2) induces growth suppression and enhances chemosensitivity of human colon cancer cells

**DOI:** 10.1186/s12935-016-0355-9

**Published:** 2016-09-29

**Authors:** Radhakrishnan Vishnubalaji, Shijun Yue, Musaad Alfayez, Moustapha Kassem, Fei-Fei Liu, Abdullah Aldahmash, Nehad M. Alajez

**Affiliations:** 1 Stem Cell Unit, Department of Anatomy, College of Medicine, King Saud University, Riyadh, 11461 Kingdom of Saudi Arabia; 2 Princess Margaret Cancer Centre, University Health Network, Toronto, ON Canada; 3 Molecular Endocrinology Unit (KMEB), Department of Endocrinology, University Hospital of Odense and University of Southern Denmark, Odense, Denmark; 4 Prince Naif Health Research Center, King Saud University, Riyadh, 11461 Kingdom of Saudi Arabia

**Keywords:** Bone morphogenetic protein2 (BMP2), Colon cancer, Chemosensitivity

## Abstract

**Background:**

Molecular profiling of colorectal cancer (CRC) based on global gene expression has revealed multiple dysregulated signalling pathways associated with drug resistance and poor prognosis. However, the role of BMP2 signaling in CRC is not fully characterised.

**Methods:**

Bioinformatics data analysis were conducted on the GSE21510 dataset. Leniviral technology was utilized to stably express BMP2 in the HCT116 CRC model. Gene expression profiling was conducted using Agilent microarray platform while data normalization and bioinformatics were conducted using GeneSpring software. Changes in gene expression were assessed using qRT-PCR. AlamarBlue assay was used to assess cell viability in vitro. In vivo experiments were conducted using SCID mice.

**Results:**

Our data revealed frequent downregulation of BMP2 in primary CRC tissues. Additionally, interrogation of publically available gene expression datasets revealed significant downregulation of BMP2 in metastatic recurrent compared to non-metastatic cancer (p = 0.02). Global gene expression analysis in CRC cells over-expressing BMP2 revealed multiple dysregulated pathways mostly affecting cell cycle and DNA damage response. Concordantly, lentiviral-mediated re-expression of BMP2 inhibited HCT116 CRC growth, sphere formation, clonogenic potential, cell migration, and sensitized CRC cells to 5-fluorouracil (5-FU) in vitro. Additionally, BMP2 inhibited CRC tumor formation in SCID mice.

**Conclusions:**

Our data revealed an inhibitory role for BMP2 in CRC, suggesting that restoration of BMP2 expression could be a potential therapeutic strategy for CRC.

**Electronic supplementary material:**

The online version of this article (doi:10.1186/s12935-016-0355-9) contains supplementary material, which is available to authorized users.

## Background

Colorectal cancer (CRC) is the third most common cancer type globally with an annual incidence of over 1.2 million new cases. It is also a leading cause of cancer-related mortality, and accounts for >600,000 cancer-related deaths each year [1–3]. Traditionally, CRC has been classified based on standardized histopathological features; more recently, molecular genotyping has also been investigated. Both technologies revealed a complex and heterogeneous nature of CRC. Several pathological mechanisms and signalling pathways have been identified as contributing to the pathophysiology of this disease, and some of these mechanisms already operate in the precancerous lesions, which has been termed the common serrated tumour pathway [4]. Among these signalling pathways, bone morphogenetic protein signalling is relevant [5].

Bone morphogenetic proteins (BMP) belong to the transforming growth factor beta superfamily (TGFβ) and are important regulators of embryogenesis, and patterning of body axes. They regulate adult tissue homeostasis through control of cell growth, apoptosis and differentiation. The biological effects of BMPs have been mostly studied in mesoderm-derived cells and tissues, and to lesser degree in epithelial cells and tissues. A number of studies have examined the association of abnormal BMP signalling with cancer initiation and/or progression. Changes in BMP signaling via the Smad cascade has been associated with a number of tumour types [6, 7]. Also, BMP-2, -4 and -7 have exhibited altered expression in many types of malignancies including breast, prostate, osteosarcoma, glioma, ovarian, pancreatic, lung, and other cancers.

Dysfunctional BMP signaling has been implicated in CRC. Mutations in BMP receptor 1A (*BMPR1A*) or its downstream effector SMAD4 have been identified in patients with juvenile polyposis (JP), a rare autosomal dominant hamartomatous polyposis syndrome with a 12 fold increased lifetime risk for development of CRC [5]. BMP signaling molecules are expressed in human CRCs, and BMP signaling has been shown to induce growth suppression through the BMPR1A and intracellular SMAD pathway [8–10].

We have previously reported the global mRNA expression profiling in CRC tissues as compared to adjacent normal mucosa, and identified several dysregulated mRNAs including down regulation of BMP2 [2]. In the current study, we examined the biological role of BMP2 in CRC. We employed lentiviral gene transfer technology to overexpress BMP2 in the human HCT116 CRC cell line, and examined its effects on cell proliferation, migration, clonogenic potential, drug resistance, and in vivo tumour growth.

## Methods

### Cells lines and tissue culture

The human colorectal cancer (HCT116) cell line was obtained and subsequently was authenticated by Genetica DNA Laboratories, Inc. Burlington, (NC, USA). Cells were maintained in DMEM supplemented with 10 % fetal bovine serum (Gibco-Invitrogen, Waltham, MA, USA) and 100 mg/l penicillin/streptomycin. All cells were maintained in a 37 °C incubator with humidified 5 % CO_2_.

### Lentiviral transduction

Lentiviral particles encoding for LV BMP2 (LP-A0241-Lv105-0200-P) or control lentiviral particles (LP-EGFP-LV105-0200) were purchased from Genecopoeia (Genecopoeia Inc., Rockville, MD, USA). One hundred fifty thousand HCT116 cells were seeded in complete DMEM in 24-well plate. Twenty-four hours later (~80 confluency), media was removed and then 20 μl of crude lentiviral particles in 500 μl of DMEM + 5 % heat-inactivated serum (Invitrogen) and 1 % Pen–Strep supplemented with polybrene (8 μg/ml; Sigma, St. Louis, MO, USA) was added to the cells. Seventy-two hours later, media was removed and transduced cells were selected with puromycin (1 μg/ml, Sigma, St. Louis, MO. USA) for 1 week or until stably transduced cells were generated.

### Gene expression microarray

RNA isolation, gene and microRNA expression analysis were performed in accordance with our previously published protocols [11]. In brief, RNA was isolated using Total RNA Purification Kit from Norgen-Biotek Corp. (Thorold, ON, Canada) and were quantified using NanoDrop 2000 (Thermo Scientific, Wilmington, DE, USA). Total RNA was labelled and then hybridized to the Agilent Human SurePrint G3 Human GE 8 × 60 k mRNA microarray chip (Agilent Technologies). All microarray experiments were conducted at the Microarray Core Facility (Stem Cell Unit, College of Medicine, King Saud University). Data were subsequently normalized and analyzed using GeneSpring 13.0 software (Agilent Technologies). Pathway analyses were conducted using the Single Experiment Pathway analysis feature in GeneSpring 13.0 (Agilent Technologies). The Benjamini-Hochberg false discovery rate (FDR) multiple testing correction method [p(corr) < 0.02] was utilized and two fold cut-off were used to enrich for significantly changed transcripts.

### mRNA validation by qRT-PCR

mRNAs expression levels were validated in LV control and LV BMP2 HCT116 cells using SYBR Green-based qRT-PCR and the Applied Biosystems ViiA™ 7 Real-Time PCR System. Two μg of total RNA was reverse transcribed using High Capacity cDNA Reverse Transcript Kit (Part No: 4368814; ThermoFisher Scientific, Waltham, MA, USA) according to the manufacturer’s protocol. Primer sequences used in the current study are listed in Table 1. The relative expression level was calculated using −ΔΔCT. GAPDH was used as an endogenous control.

**Table 1:** List of CYBR green primers used in current study

No	Name	Sequence
1	CASP10	F 5′ GAAGCCTTACCGCAGGAGTC 3′
		R 5′ GTGCACCATTTGTGGCTCTG 3′
2	HDAC4	F 5′ ACTGGTACGGGAAAACGCAG 3′
		R 5′ TTTGGCGTCGTACATTCCCA 3′
3	MAPK9	F 5′ TGGGCTACAAAGAGAACGTTGAT 3′
		R 5′ AAGGTCGCGGGGAAGGATA 3′
4	MDM2	F 5′ AGGAGATTTGTTTGGCGTGC 3′
		R 5′ TGAGTCCGATGATTCCTGCTG 3′
5	WNT7A	F 5′ ATGCCCGGACTCTCATGAAC 3′
		R 5′ GTTCTCCTCCAGGATCTTTCGG 3′
6	WNT3	F 5′ GGACAAAGCTACCAGGGAGT 3′
		R 5′ CTGCACATGAGCGTGTCACT 3′
7	WNT4	F 5′ CATGAGTCCCCGCTCGTG 3′
		R 5′ CCAGGTACAGCCAGTTGCTC 3′
8	SMAD4	F 5′ GGATACGTGGACCCTTCTGG 3′
		R 5′ TGTGCAACCTTGCTCTCTCAA 3′
9	TERT	F 5′ GGCACGGCTTTTGTTCAGAT 3′
		R 5′ TCCGGGCATAGCTGGAGTAG 3′
10	AFT3	F 5′ GTGAGTCCTCGGTGCTCG 3′
		R 5′ GCATCATTTTGCTCCAGGCT 3′
11	HOMEZ	F 5′ CTGGACTGCGCTATCTCTGAAG 3′
		R 5′ TGAACTACTGAGACCGCTGG 3
12	FADD	F 5′ GGGAAGAAGACCTGTGTGCAG 3′
		R 5′ GAGCCAGCCTTCTCCAATCT 3′
13	BCL2	F 5′ AGATTGATGGGATCGTTGCCT 3′
		R 5′ AGTCTACTTCCTCTGTGATGTTGT 3′
14	BIRC5	F 5′ AGCCAAGAACAAAATTGCAAAGG 3′
		R 5′ CGCACTTTCTCCGCAGTTTC 3′
15	E2F1	F 5′ AACTGACCATCAGTACCTGGC 3′
		R 5′ GGGATTTCACACCTTTTCCTGG 3′
16	E2F2	F 5′ AGGAGCAGACAGTGATTGCC 3′
		R 5′ GGTTGTCCTCAGTCCTGTCG 3′
17	BMP2	F 5′ GGAACGGACATTCGGTCCTT 3′
		R 5′ CACCATGGTCGACCTTTAGGA 3′
18	GAPDH	F 5′ CTGGTAAAGTGGATATTGTTGCCAT 3′
		R 5′ TGGAATCATATTGGAACATGTAAACC 3′

### Measurement of cell viability, clonogenic and sphere formation

The viability of LV control and LV BMP2 HCT116 cells was determined using alamarBlue assay as previously described [12]. All assays were carried out with appropriate controls. Briefly, 10,000 cells were cultured in a 96-well plate and cell viability was measured at the indicated time points by adding 10 % volume alamarBlue assay reagent and measuring absorbance at 570 λ. The colony forming ability of HCT116 cells transduced with BMP2 was determined using clonogenic assay as previously described [12, 13]. Briefly, LV control or BMP2 HCT116 cells were seeded in 12-well plates in different serial dilution (1:2–1:64). Initial seeding density was 0.015 × 10^6^ cells per well, and incubated at 37 °C under 5 % CO_2_ for 10 days. The plates were then washed and stained with Diff-Quik stain set (Siemens Healthcare Diagnostics), and the plates were scanned and number of colonies were observed under microscope. The fraction of surviving cells was estimated by comparing BMP2 to LV control cells. The experiment was done twice in duplicate. Furthermore, the clonogenic assay was conducted to examine the effect of 5-fluorouracil on cell growth and proliferation in both cells. A total of 1 × 10^6^ cells were seeded in T25 flask. After 48 h of exposure to 1.5 µM of 5-fluorouracil, the cells were trypsinized and reseeded in 12-well plates as described above to observe the effect of the drug.

Multicellular tumor spheroids were produced in 60 mm low cell binding dishes (Nunc; ThermoFisher Scientific). LV control or BMP2 HCT116 cells were trypsinized from monolayers and transferred to the low cell binding dishes. The formation of tumor spheroids was performed with 10,000 cells. On day 10, established spheroids were analysed.

### Cell migration and proliferation

Real-time measurement of LV control and BMP2 HCT116 cell migration and proliferation was executed using the xCELLigence RTCA DP device (ACEA Biosciences, San Diego, CA). For migration study, cells were starved for 24 h in 1 % serum media, followed by seeding 0.08 × 10^6^ cells per well in 16-well microelectronic sensor plate pre-coated with fibronectin (1:500), two chamber trans-well plates (CIM-plate insert; ACEA Bioscience) containing the respective serum conditions. Medium containing 10 % serum (chemo-attractant) and 1 % serum (negative control) was added to the bottom wells.

For proliferation assay, cells were seeded (0.04 × 10^6^ cells/well) in two chamber plates (E-plate insert; ACEA Bioscience). Proliferation and migration of cells was measured from the interaction of cells with the electrodes on the bottom surface of top chamber and represented as a change in cell index (CI). The electrical impedance was captured every 15 min for an experimental duration of ~68 h. The rate of migration and proliferation is expressed as the CI or the change in electrical impedance at each time-point. The Cell Index at each time point is defined as (Rn-Rb)/4.6, where Rn is the cell-electrode impedance of the well when it contains cells and Rb is the background impedance of the well with the media alone. Values are expressed as the mean ± SEM of the 3 replica wells.

For conventional migration, the BD transwell migration system with 8 µ pore size was utilized as previously described [14]. Inserts were placed in a 24-well plate, and 1.56 × 10^5^ cells in 1 % serum were added to the top of the chamber, and 10 % serum added to the bottom chamber. Hundred hours later, inserts were fixed and stained with SIEMENNS DIFF-QUICK stain set (Siemens Healthcare Diagnostics), and the number of migrating cells was counted using a light microscope.

### Measurement of apoptosis

Fluorescence-based apoptosis was determined in cells after exposure to different concentration of 5-fluorouracil, using acridine orange and ethidium bromide (AO/Etbr) staining method. After treatment, the LV control and BMP2 HCT116 cells were stained with dual fluorescent staining solution (1 µl) containing 100 µg/ml AO and 100 µg/ml EB (AO/EB, Sigma, St. Louis, MO). Cells were washed twice with PBS and were gently mixed with AO/EB (1:100) dye solution for 1 min; afterwards, the cells were observed and photographed under a Nikon Eclipse Ti fluorescence microscope. Cells cultured without drug were considered as experiment control. Acridine Orange/Ethidium Bromide Staining uses combination of two dyes to visualize cells with aberrant chromatin organization. The differential uptake of AO/EB allows the identification of viable and non-viable cells. Particularly, Acridine Orange was used to visualize the number of cells which has undergone apoptosis.

### In vivo tumorigenicity assay in SCID mice

In vivo tumor formation was carried out as previously described [15]. Briefly, 6- to 8-week-old severe combined immunodeficient (SCID) mice were utilized for the xenograft experiments. Ten million LV control or LV BMP2 HCT116 cells were suspended in PBS and subcutaneously injected into the right flank of SCID mice. Tumor volume was measured twice weekly using a caliper, and tumor volume was calculated as (tumor length × width^2^)/2. At the end of the experiment, tumors were excised, fixed in 10 % buffered formalin, and embedded in paraffin and sectioned. Sections were the stained with haematoxylin and eosin.

### Immunohistochemistry

We used commercially available anti-bcl-2 (124) mouse monoclonal primary antibody (790-4464; Ventana; Roche) and anti-Ki67 antibody rabbit polyclonal (ab15580; Abcam) in 1:200 dilution to stain the tumor section. Four-micron-thick sections were immunostained for Bcl-2 and Ki67 protein using the immunohistochemical (IHC) staining protocol optimized in King Khalid university hospital, King Saud University, Riyadh. The IHC staining was carried out using a BenchMark XT fully automated IHC/ISH staining instrument (Ventana Medical system Inc, Tucson, Arizona, USA), as per manufacturer’s protocol using proprietary reagents. Briefly, slides were deparaffinized on the automated system with EZ Prep solution (Ventana). Enzymatic retrieval was used with protease 1 solution (Ventana). The detection system used was the ultraView Universal DAB Detection Kit (760-500; Ventana; Roche), and slides were then counterstained with hematoxylin and mounted.

### Statistical analysis

Statistical analyses and graphing were performed using Microsoft Excel 2010 and GraphPad Prism 6.0 software (GraphPad, San Diego, CA, USA). P values were calculated using the two-tailed *t* test.

## Results

### BMP2 is downregulated in CRC and its overexpression reduces HCT116 cell growth, migration, sphere formation and colony formation

**Fig. 1:**
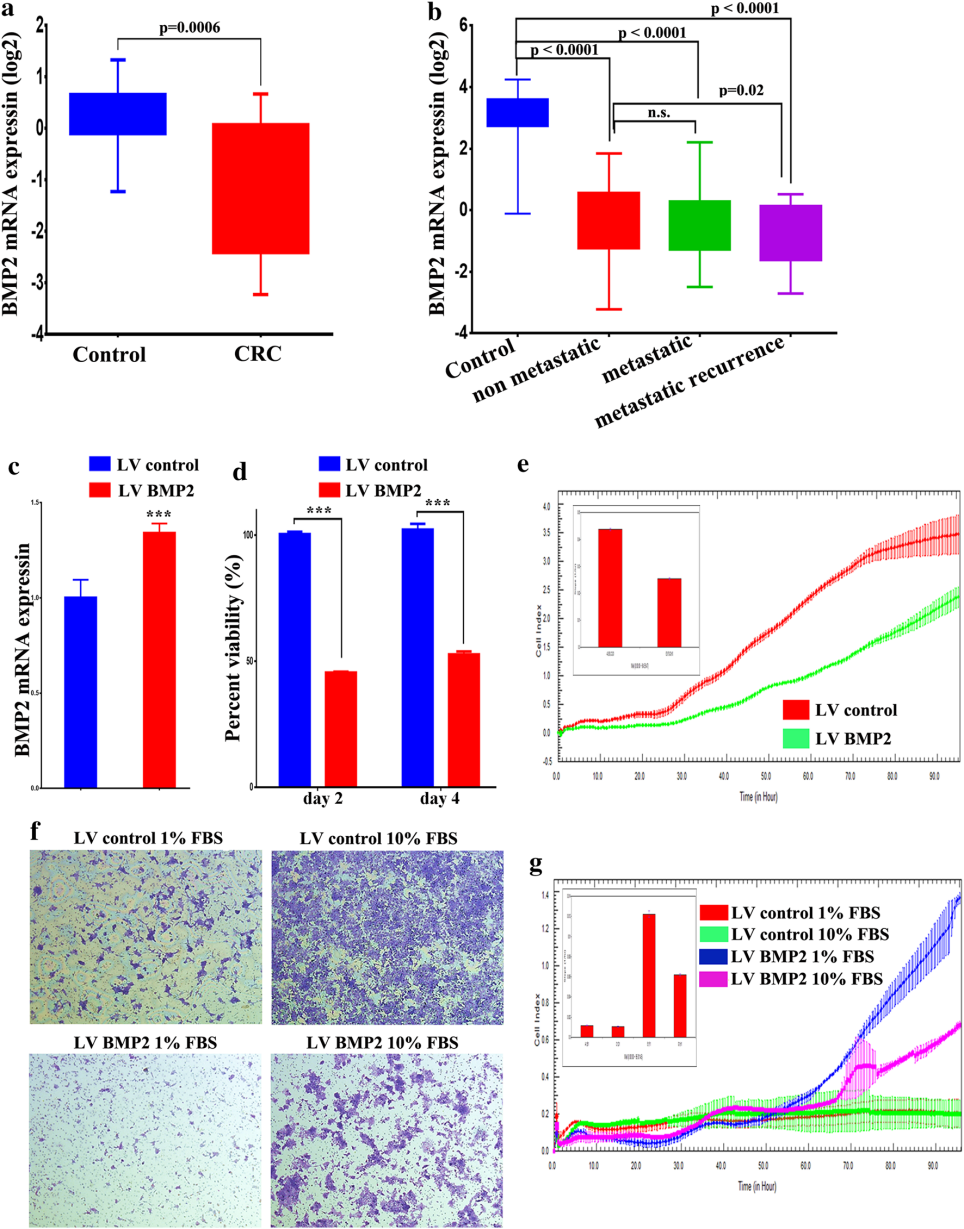
BMP2 is downregulated in CRC and it suppresses CRC cell proliferation and migration. **a** Expression of BMP2 in CRC (Log2) compared to adjacent normal tissue based on microarray data. Data are presented as mean ± S.E., n = 13. **b** Expression of BMP2 in control (n = 25), non-recurrent (n = 76), metastatic (n = 23), and metastatic recurrent (n = 24) from the GSE21510 CRC dataset. **c** qRT-PCR quantification of BMP2 expression in BMP2 HCT116 compared to LV control cells. Data are presented as mean ± S.D., n = 3. **d** Lentiviral-mediated re-expression of BMP2 in HCT116 cells reduces their cell viability. **e** Real time proliferation assay revealed significant decrease in the proliferation of BMP2 HCT116 compared to LV control cells in a time-dependent manner. **f**, **g** Conventional and real time migration assay showing significant inhibition of cell migration in the BMP2 HCT116 compared to LV control cells. The two-tailed t-test was used to compare different treatment groups. ***p < 0.0005

Global mRNA gene expression profiling of CRC tissue and adjacent normal mucosa revealed decreased levels of BMP-2 gene expression (Fig. 1a) [2]. Follow up bioinformatics analysis of CRC gene expression data using the GEO database (GSE21510) revealed similar pattern of down regulation of BMP-2 gene expression in CRC compared to normal tissues, and this was also observed in metastatic and metastatic recurrent CRC lesions, suggesting that loss of BMP2 is an unfavourable event in CRC pathogenesis and progression (Fig. 1b). Lentiviral-mediated stable overexpression of BMP2 reduced viability of HCT116 CRC cells in vitro (Fig. 1c, d). Adding exogenous recombinant BMP2 to HCT116 cells led to similar results (Additional file 1: Figure S1). Concordantly, real time proliferation assay revealed striking decrease in the proliferation of LV-BMP2-HCT116 cells compared to LV control cells in a time dependent manner (Fig. 1e). Similar inhibitory effects were also observed on cell migration toward media containing 10 % FBS in the LV-BMP2-HCT116 compared to LV control cells employing two independent assays: transwell migration assay (Fig. 1f) and microelectronic sensor plate assay (Fig. 1g), implicating a role for BMP2 in proliferation as well as in migration.

**Fig. 2:**
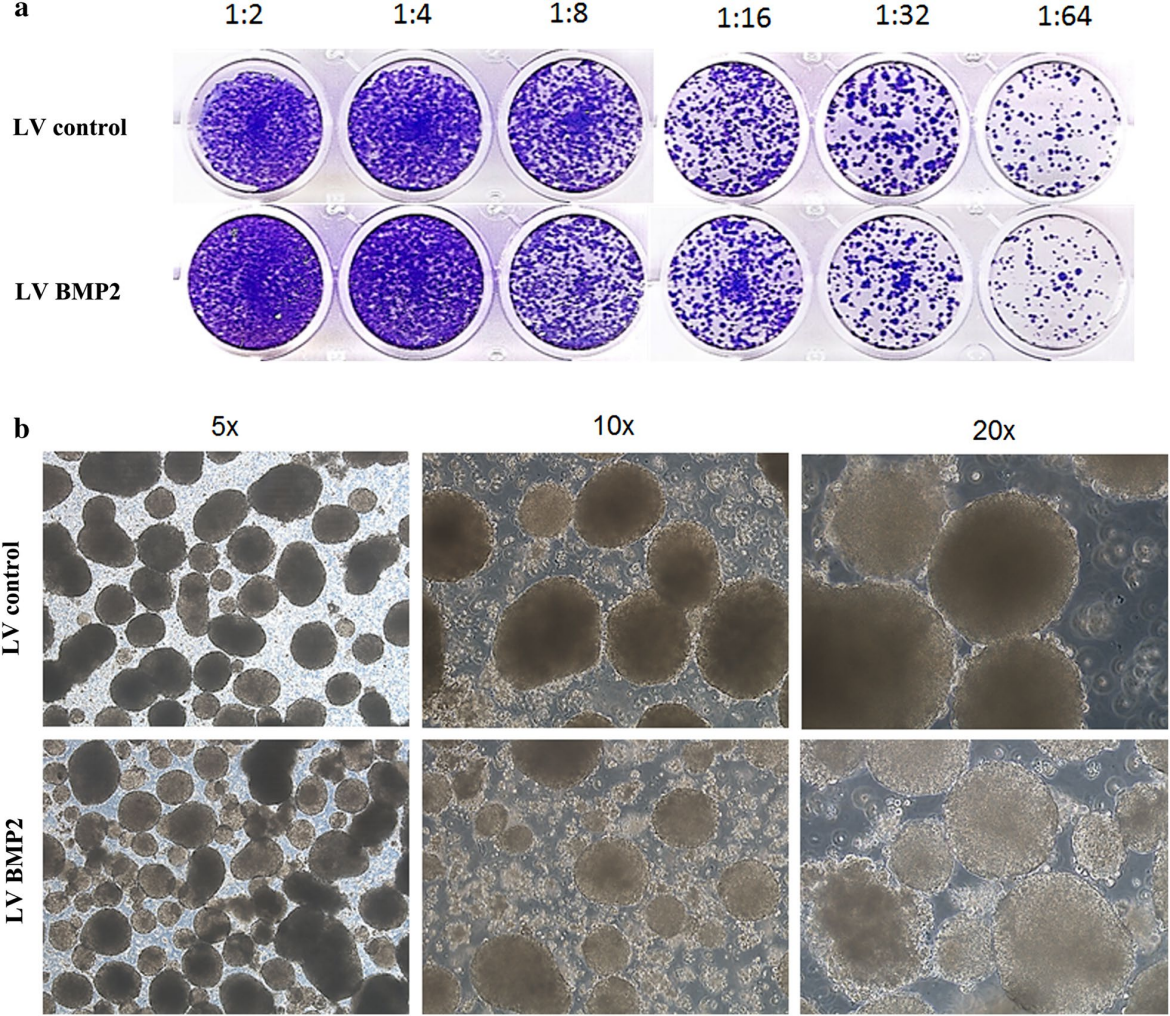
BMP2 reduces CRC colony and sphere formation in vitro. **a** Clonogenic assay showing remarkable reduction in the colony forming capability of BMP2 HCT116 cells compared to LV control cells. Plates were stained with Diff-Quik stain set on day 10. Wells are representative of two independent experiments for each condition. **b** Inhibition of sphere formation by BMP2 in the HCT116 CRC model

In agreement with proliferation data, the clonogenic assay revealed fewer colonies in the LV-BMP2-HCT116 compared to LV control cells (Fig. 2a), suggesting an inhibitory effect of BMP2 on colony forming unit in the HCT116 model. We subsequently assessed the ability of those cells to form spheres when cultured in low adherence plates. The control tumor formed spheres with compact and clear rounded edges, while the LV-BMP2 tumour-derived spheres were less compact and have irregular edges (Fig. 2b).

### Dysregulated genetic pathways in LV-BMP2-HCT116 cells

**Fig. 3:**
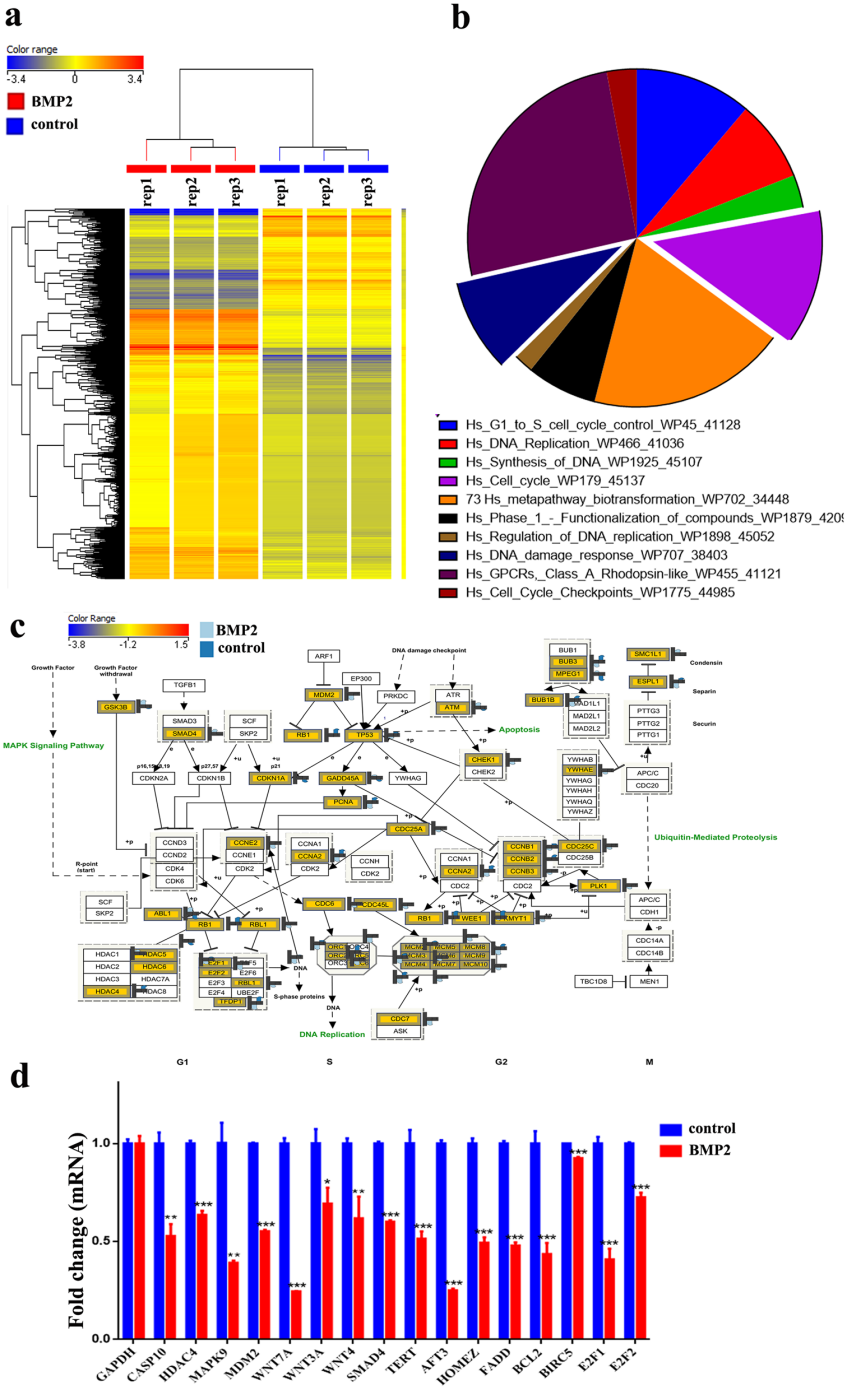
BMP2 regulated multiple cellular processes in HCT116 cells. **a** Hierarchical clustering of BMP2 HCT116 vs LV control HCT116 cells based on differentially expressed mRNA levels. Each* column* represents one replica and each* row* represents a transcript. Expression level of each gene in a single sample is depicted according to the colour scale. **b** Pie chart illustrating the distribution of the top 10 pathway designations for the differentially expressed genes in BMP2 HCT116 cells. The pie size corresponds to the number of matched entities. **c** The cell cycle pathway is illustrated in *panel c*. **d** The expression levels of selected genes from the microarray data were validated using qRT-PCR in BMP2 HCT116 cells. Data are presented as mean ± S.D, n = 3

To unravel the molecular processes regulated by BMP2, we performed global mRNA expression profiling on LV-BMP2-HCT116 and LV-Control cells. As shown in Fig. 3a, hierarchical clustering based on differentially-expressed mRNAs revealed clear separation between the two groups. We identified 11,950 differentially-expressed transcripts in LV-BMP2-HCT116 cells [>2.0 fold change (FC), p(corr) < 0.02; Additional file 2: Table S1]. The distribution of the top 10 enriched pathways of the differentially-expressed genes in LV-BMP2-HCT116 cells are shown in Fig. 3b, which included cell cycle, DNA replication, and DNA damage response pathways. Illustration of the cell cycle pathway is presented in panel Fig. 3c and Additional file 3: Figure S2. We validated the expression level of a panel of selected genes using qRT-PCR: CASP10, HADAC4, WNT3, MAPK9, MDM2, WNT7A, WNT3A, WNT4, SMAD4, TERT, AFT3, HOMEZ, FADD, BCL2 and E2F2 (Fig. 3d).

### BMP2 increases CRC cell sensitivity to 5-fluorouracil

**Fig. 4:**
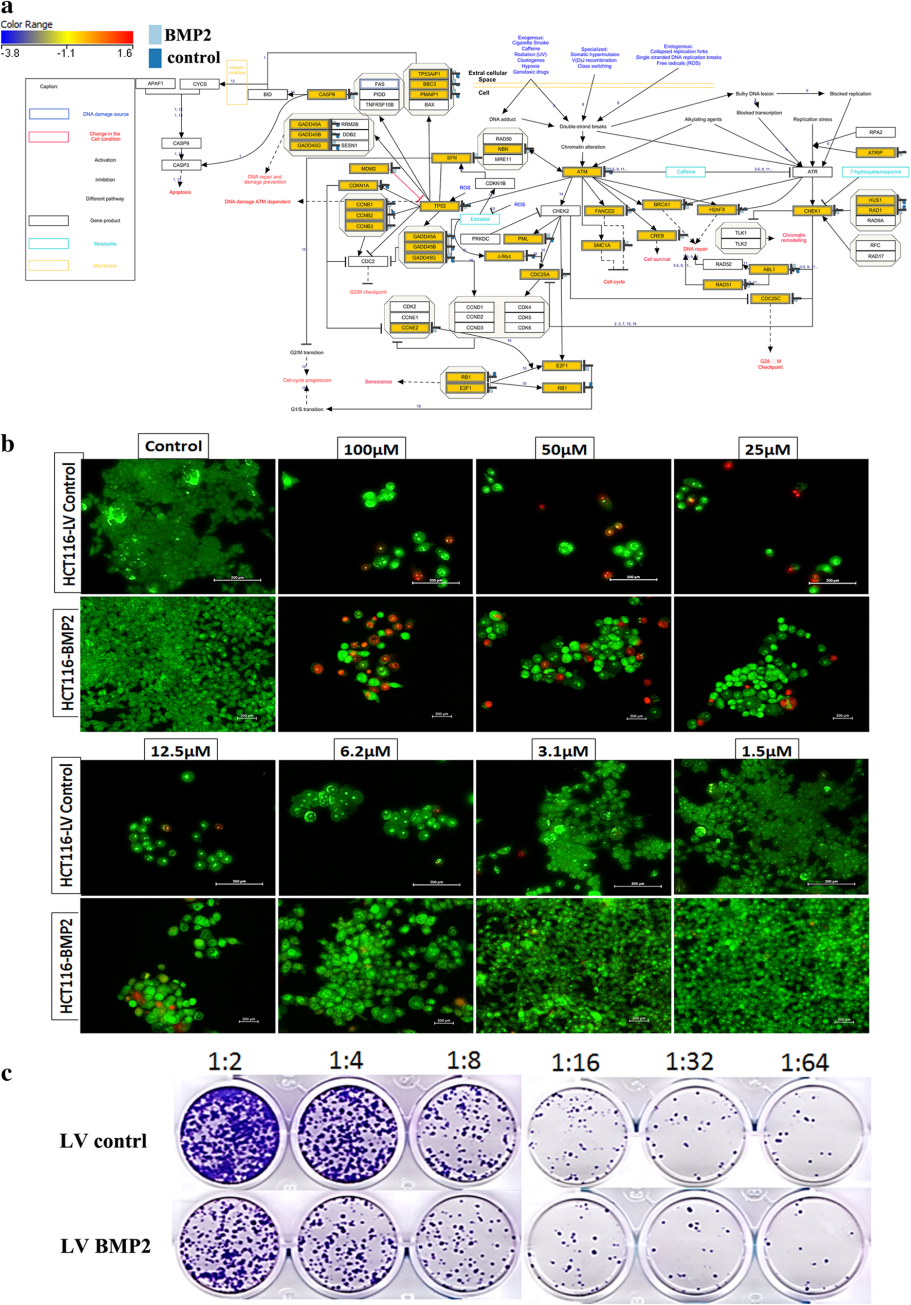
BMP2 sensitize CRC cells to 5-fluorouracil. **a** Illustration of the DNA damage response pathway based on microarray data with matched entities highlighted. **b** Representative fluorescence images of BMP2 and LV control HCT116 cells [±different concentration (1.5–100 μM) 5-fluorouracil]. Cells were stained with acridine orange/ethidium bromide to detect apoptotic (cells with green condensed chromatin) and necrotic cells (red) **c.** Representative clonogenic assay showing reduced clonogenicity of BMP2 HCT116 compared to LV control HCT116 cells (±1.5 μM 5-fluorouracil). Plates were stained with Diff-Quik stain set on day 10

Pathway analysis of differentially-expressed genes in LV-BMP2-HCT116 cells revealed enrichment in genes associated with response to DNA damage (Fig. 3b), suggesting that BMP2 may sensitize cancer cells to DNA damage-inducing agents. Illustration of the DNA damage response pathway is presented in Fig. 4a and Additional file 4: Figure S3. To test this hypothesis, LV-BMP2-HCT116 and LV control HCT116 cells were incubated in the presence of 5-FU (1.5–100 μM), then assessed for apoptotic/necrotic response. 5-FU concentrations exceeding >3.1 µM were highly toxic; whilst lower concentrations (<3.1 µM) induced more apoptosis in the BMP2-HCT116 compare to LV Control HCT116 cells on day 5 (Fig. 4b). As expected, fewer colonies were observed in the LV-BMP2-HCT116 cells in the presence of 5-FU (1.5 μM) compared to LV control-HCT116 cells (Fig. 4c).

### BMP2 suppresses tumor growth in vivo

**Fig. 5:**
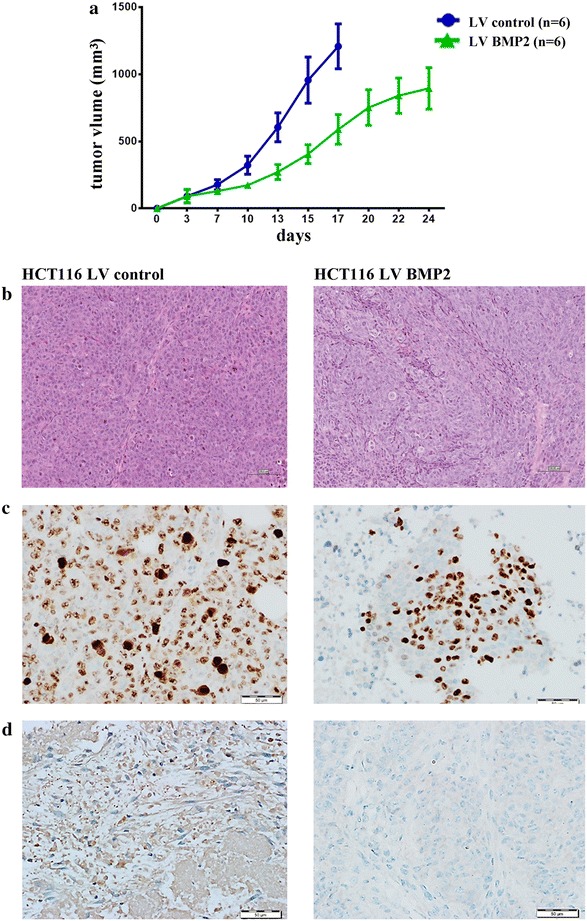
BMP2 expression suppresses CRC growth in vivo. **a** Tumour formation in SCID mice after subcutaneous injection of HCT116 cells stably-expressing BMP2 or LV control cells. Data are presented as mean (tumor volume) ± S.E., n = 6. Two-way ANONA analysis was used to compare the two growth curves. **b** Representative histopathological examination of xenograft tumors from BMP2 and control HCT116 cells. FFPE sections were stained with haematoxylin and eosin stain. (Bar = 100 μm). Expression of MKI67 (**c**) and BCL2 (**d**) in xenograft tumors from BMP2 and control HCT116 cells is shown

To further understand the biological significance of BMP2 on CRC tumorogenesis in vivo, LV-BMP2-HCT116 or LV control HCT116 cells were implanted subcutaneously into immune deficient SCID mice, and the volume of formed tumors was subsequently determined. Significant reduction in tumor growth was observed for the LV-BMP2-HCT116 tumours, corroborating the in vitro inhibitory observations of BMP2 (Fig. 5a). Histological examination of tumor xenograft tissues revealed a higher degree of cell death (necrosis and apoptosis), and a reduced frequency of mitotic events in the LV-BMP2-HCT116 group (Fig. 5b).

## Discussion

Identifying signalling pathways that are associated with cancer is important in order to identify novel targets for therapy. In the current study, we have demonstrated that enhanced expression of BMP2 in CRC cells reduced their growth, enhanced apoptosis and decreased tumor development in vivo. Given our previous demonstration that CRCs expressed lower levels of BMP2 [2]; the more pronounced downregulation of BMP2 in metastatic-recurrent patients from the GSE21510, combined with the current data, this all indicate that BMP2 and BMP signalling are important regulators of CRC development and progression. Although the exact molecular mechanism leading to BMP2 downregulation in CRC is still unknown, genomic deletion, DNA methylation or epigenetic regulation could potentially play a role in BMP2 downregulation in CRC. Downregulation of BMP2 was also observed in several CRC cell lines (Additional file 5: Figure S4).

We employed HCT116 as a cell model for CRC; overexpression of BMP2 inhibited CRC proliferation, migration, colony and spheroid formation and in vivo tumor growth, demonstrating a tumor suppressive role for BMP2. Restitution of BMP2 expression also restored sensitivity to 5-FU. Molecular signature of HCT116 cells overexpressing BMP2 revealed multiple altered genetic pathways including cell cycle, DNA replication, DNA damage response, and WNT signalling, which could provide the biological explanations for the observed association between BMP2 status and CRC. Interestingly, our recent data revealed a significant role for TGF-beta in CRC, as pharmacological inhibition of TGF-beta pathway inhibited CRC growth in vitro [2]. Similar to our findings, Zhang et al. also recently reported an inhibitory role for BMP2 in governing the proliferation and aggressive behavior of human CRC cells [16].

Genetic studies showed mutations in the TGF-β signaling pathway to increase the mortality risk in CRC patients [17]. Additionally, a number of other studies have reported that BMP2 expression is dysregulated in CRC. BMPR1a, BMPR1b, BMPR2, phosphorylated Smad1, and Smad4 are expressed in mature colon cells in normal adult human and mouse colon [8]. Several members of the BMP family (BMP2, BMP3 or BMP7) are downregulated in CRC, and have been shown to promote apoptosis, differentiation, and inhibit proliferation [8–10, 16]. Our study corroborates these findings; identifying a specific role for BMP2 in CRC. Furthermore, we provide evidence suggesting that targeting BMP2 signalling could be a possible approach to limit CRC tumour growth.

While our study suggest a suppressive role for BMP2 in CRC, the precise role for the BMP signalling in tumour development is controversial since it has been reported to either promote or inhibit tumorogenesis [7]. Therefore it is possible that the effect of BMP signalling is dependent on tumour type as well as the status of other pathways in different cancers. For example, Alarmo et al. reported that BMP7 can either promote or inhibit carcinogenesis, depending on dose, type of cell or tissue origin, and the environmental niche, reflecting the complexity of networks and pathways involved in cancer development and progression [18].

## Conclusions

We propose that loss of BMP2 leads to deregulation in multiple signaling pathways involving cell cycle, DNA replication, and DNA damage response resulting in CRC development, progression and possibly resistance to chemotherapy. Targeting the BMP2 pathway may therefore offer potential therapeutic benefit in CRC therapy.

## Additional files

10.1186/s12935-016-0355-9 Recombinant BMP2 inhibit HCT116 cell growth in vitro. HCT116 cells were treated with 500 ng/ml recombinant BMP2 and cell viability was assessed on day 4 using the alamarblue assay. Data are presented as mean ± S.D. from two experiments, n = 8.
10.1186/s12935-016-0355-9 Differentiatlly expressed genes (>2.0 FC) between HCT116 LV BMP2 and HCT116 LV control cells.
10.1186/s12935-016-0355-9 Illustration of the cell cycle pathway.
10.1186/s12935-016-0355-9 Illustration of the DNA damage response pathway based on microarray data with matched entities highlighted.
10.1186/s12935-016-0355-9 BMP2 expression in CRC cell lines from the Cancer Cell Line Encyclopedia. Data are presented as mRNA expression (Log2).

## Abbreviations

CRC: colorectal cancer
RNA: ribonucleic acid
mRNA: messenger RNA
BMP2: bone morphogenetic protein 2
qRT-PCR: quantitative reverse transcription polymerase chain reaction
TGF-beta: transforming growth factor-β
GAPDH: glyceraldehyde 3-phosphate dehydrogenase
ABI: applied biosystem
SCID: severe combined immunodeficient
TGFβ: transforming growth factor beta superfamily
DMEM: Dulbecco’s modified eagle’s medium
FDR: false discovery rate
LV: lentiviral
CI: cell index
FBS: fetal bovine serum
