# Supplementary figures and images for: Bone morphogenetic protein 2 (BMP2) induces growth suppression and enhances chemosensitivity of human colon cancer cells
Source: Cancer Cell Int. 2016 Sep 29;16:77. doi: 10.1186/s12935-016-0355-9 (PMC5043592; doi:10.1186/s12935-016-0355-9)

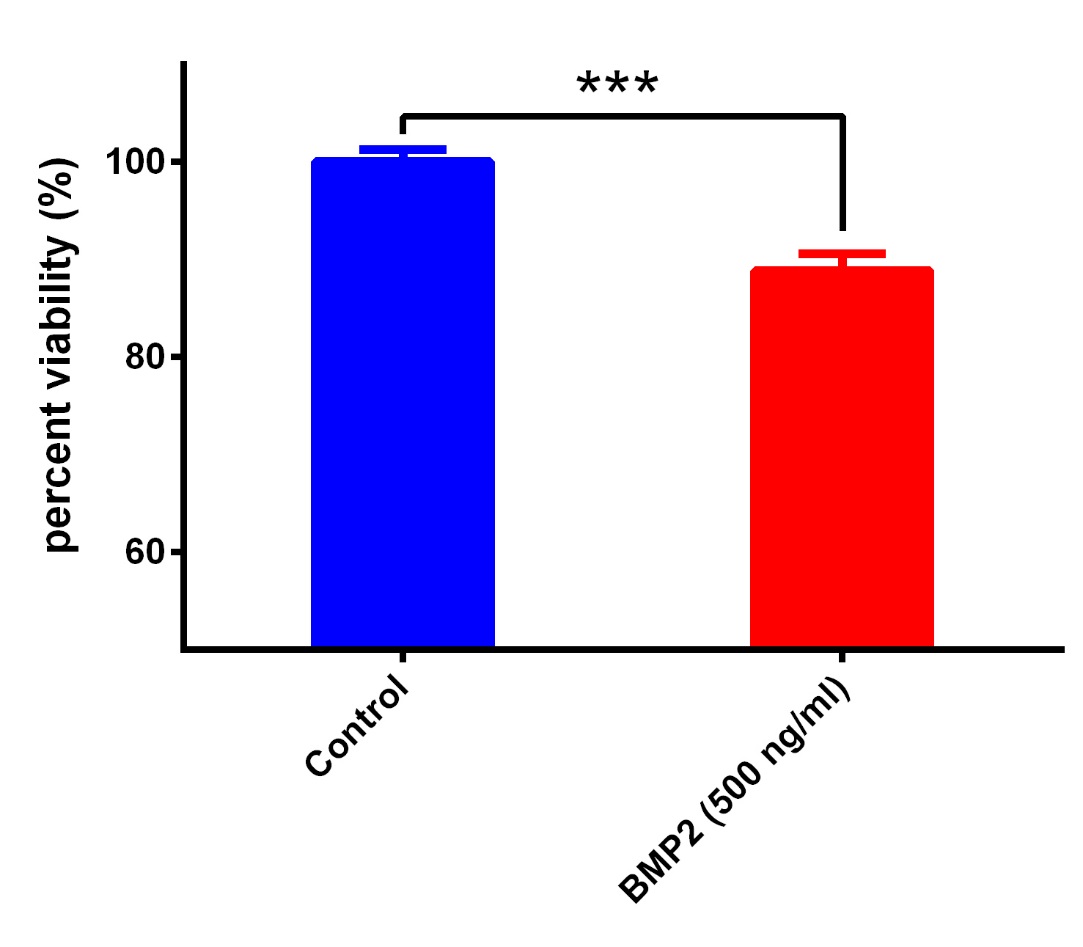


**Additional figure S1.**

Supplement: Supplementary file 1 — 10.1186/s12935-016-0355-9 Recombinant BMP2 inhibit HCT116 cell growth in vitro. HCT116 cells were treated with 500 ng/ml recombinant BMP2 and cell viability was assessed on day 4 using the alamarblue assay. Data are presented as mean ± S.D. from two experiments, n = 8. [file 12935_2016_355_MOESM1_ESM.docx]
