# Supplementary material for: Bone morphogenetic protein 2 (BMP2) induces growth suppression and enhances chemosensitivity of human colon cancer cells
Source: Cancer Cell Int. 2016 Sep 29;16:77. doi: 10.1186/s12935-016-0355-9 (PMC5043592; doi:10.1186/s12935-016-0355-9)
Supplement: Supplementary file 5 — 10.1186/s12935-016-0355-9 BMP2 expression in CRC cell lines from the Cancer Cell Line Encyclopedia. Data are presented as mRNA expression (Log2). [file 12935_2016_355_MOESM5_ESM.pdf]

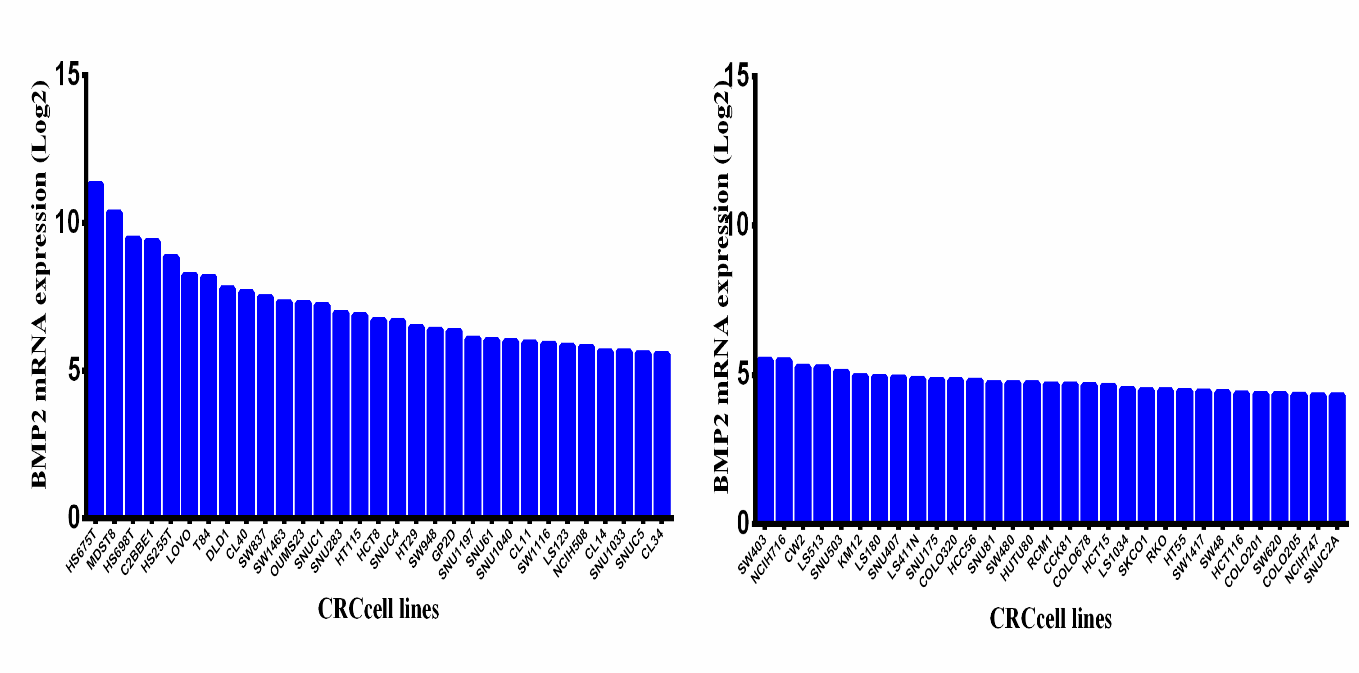

Supplementary figure 4. BMP2 expression in CRC cell lines from the Cancer Cell Line Encyclopedia. Data are presented as mRNA expression (Log2).
